# Barriers and carriers: a multicenter survey of nurses’ barriers and facilitators to monitoring of nurse‐sensitive outcomes in intensive care units

**DOI:** 10.1002/nop2.85

**Published:** 2017-05-27

**Authors:** Dewi Stalpers, Maartje L.G. De Vos, Dimitri Van Der Linden, Marian J. Kaljouw, Marieke J. Schuurmans

**Affiliations:** ^1^ St. Antonius HospitalNieuwegeinThe Netherlands; ^2^ Quality and Safety DepartmentAmphia HospitalBredaThe Netherlands; ^3^ Institute of PsychologyErasmus UniversityRotterdamThe Netherlands; ^4^ Dutch Healthcare AuthorityUtrechtThe Netherlands; ^5^ Department of RevalidationNursing Science & SportsUniversity Medical Centre UtrechtUtrechtthe Netherlands; ^6^ University of Applied SciencesUtrechtThe Netherlands

**Keywords:** attitude, barriers, behaviour, intensive care units, knowledge, nurses, quality indicators, questionnaires

## Abstract

**Aim:**

To identify nurses’ barriers and facilitators to monitoring of nurse‐sensitive outcomes in intensive care units (ICUs), and to explore influential nurse characteristics and work environment factors.

**Design:**

A cross‐sectional survey in three Dutch ICUs between October 2013 ‐ June 2014.

**Methods:**

A questionnaire with questions regarding facilitators and three types of barriers: knowledge, attitude and behaviour. The Dutch Essentials of Magnetism II was used to examine work environments.

**Results:**

All 126 responding nurses identified pressure ulcers and patient satisfaction as outcomes that are nurse‐sensitive and nurses’ full responsibility. Lack of time (behaviour) was perceived as the most prominent barrier, followed by unfamiliarity with mandatory indicators (knowledge), and unreliability of indicators as benchmark data (attitude). Education and clear policies were relevant facilitators. Of nurse characteristics, only regularity of shifts was related to perceived attitude related barriers. The work environment factor “clinical autonomy” was potentially associated with behaviour related barriers.

## 1 INTRODUCTION

Nurses are first in the line of duty when it comes to the provision of care to patients in hospitals, as they are the only health care professionals present at patients’ bedside 24 hr a day. Despite the high number of nurses in health care settings and their importance in delivering good patient care, the measurement of nursing performance remains a difficult issue (Kurtzman, Dawson & Johnson, 2008). Traditionally, nurses are known to care for and nurture patients based on intuition and nursing skills; little focus on measuring the effects of a nurse's care on patient outcomes. Florence Nightingale was the first to acknowledge the importance of collecting data and its relation to the improvements of health care outcomes (Ellis, 2008). Nowadays, nurse‐sensitive outcomes are used as measures to quantify care that is provided and influenced by nurses (Maas, Johnson & Moorehead, 1996). Nurse‐sensitive outcomes (NSOs) are defined as “those outcomes that are relevant, based on nurses’ scope and domain of practice, and for which there is empirical evidence linking nursing inputs and interventions to the outcome”(Doran, 2011). Frequently mentioned examples of NSOs are pressure ulcers, patient falls and health care‐associated infections (Montalvo, 2007; Needleman, Kurtzman & Kizer, 2007). In the Netherlands, hospitals are required to report several types of NSOs to the Dutch Health Care Inspectorate, including delirium, malnutrition, pain and pressure ulcers (Inspectie voor de Gezondheidszorg 2012).

### 1.1 Background

NSOs are referred to as quality indicators and can be used for both external as well as internal purposes; in addition to their use as quality measurement tools for benchmarking hospitals, NSOs are used internally identifying areas in need of and practices for improving nursing professional care (Montalvo, 2007). It is important that nurses themselves recognize the relevance of NSOs and show their commitment to the collection of NSO data, for example by optimizing their screening activities in order to routinely gather data on NSOs. While screening activities should be an integral part of nursing practice, several studies published in the last 20 years indicate that NSO related screening processes are often suboptimal. In their study including all hospitals in the Netherlands, Leistra et al. (2014) reported an average screening percentage of 72% with regard to the screening of malnutrition, one of the mandatory nurse‐sensitive indicators. Ely et al. (2004) surveyed nearly one thousand ICU professionals and found that only 40% of nurses were routinely screening for delirium, with a mere 16% of them utilizing a formal assessment tool.

It has been previously suggested that nurses experience various barriers to the collection and completion of NSO data. Lack of time, inadequacy of measurement tools, and workload were demonstrated to be important barriers. These factors have been linked to specific NSOs, such as pressure ulcers (Strand & Lindgren, 2010), malnutrition (Leistra et al., 2014), delirium (El Hussein, Hirst & Salyers, 2014), and pain (Wang & Tsai, 2010). However, there is limited evidence of barriers to the overall use and monitoring of NSOs. The framework of Cabana et al. (1999) proposes that a wide spectrum of barriers, including barriers related to knowledge, attitude and behaviour should be assessed in order to realize the widespread behaviorial change in health care. This study was designed to assess barriers in nurses’ knowledge, attitude and behaviour to a range of NSOs, in order to give a general overview of the perceived barriers to the monitoring of NSOs. This study focused on nurses in the intensive care unit (ICU) setting as complications and adverse outcomes of care, such as NSOs are prominently present in this type of high‐risk unit (Singer et al., 2009). Besides barriers, nurse characteristics (e.g., age, educational level) and factors in nurses’ work environment (e.g., nurse‐physician relationship, staffing) are also potentially relevant in relation to nurses’ abilities to provide a high quality of care with regard to NSOs (Kane, Shamliyan, Mueller, Duval & Wilt, 2007; Stalpers, De Brouwer, Kaljouw & Schuurmans, 2015). The research questions addressed are:

- What are the barriers and facilitators to monitoring of NSOs as perceived by nurses working in ICU?
- How do nurse characteristics and factors in the work environment of ICU nurses relate to perceived barriers to NSO monitoring?

## 2 THE STUDY

### 2.1 Design

A cross‐sectional multicenter survey study in intensive care units (ICUs) was performed. Data were collected by means of a questionnaire, aimed at answering the research questions as described above (McLeod, 2014). The questionnaire included predefined statements on three types of barriers: knowledge, attitude and behaviour and facilitators to the monitoring of nurse‐sensitive outcomes (NSOs), and close‐ended questions regarding nurses’ work environment.

### 2.2 Data collection

The study was conducted in the ICUs of three teaching hospitals located in different geographical areas in the Netherlands. These hospitals were previously pilot testing hospitals for the development of the Dutch Essentials of Magnetism II instrument (De Brouwer, Kaljouw, Kramer, Schmalenberg & Van Achterberg, 2014). The ICUs labelled as level 3 ICUs, representing the highest level of ICU care in the Netherlands (Nederlandse Vereniging voor Anesthesiologie 2006), had 12 to 24 licensed beds for adult patients.

The sample consisted of the staff nurses who were active in nursing practice during the study period from October 2013 to June 2014; including scholars working more than 6 months in the ICU. Nurses with temporary contracts and staff nurses not participating in direct patient care (e.g., team leaders) were excluded. All 283 staff nurses received a paper‐based questionnaire which was anonymous and voluntarily. The questionnaires could be returned in a sealed box which was placed in each of the three ICUs. The study contact person in each of the three units (ICU nurses with an additional research education) motivated nurses to fill in the questionnaire. The primary researcher was present in the ICUs during the data collection period and sent several email reminders to the nurses.

### 2.3 Questionnaire

The first part of the questionnaire referred to the demographic features of nurses; including age, gender, years of nursing experience, years of experience as an ICU nurse, highest level of education (Associate Degree in Nursing versus Bachelor Degree in Nursing or higher), full‐time versus part‐time employment status (32 or more hr/week versus less than 32 hr/week) and regularity of shift schedules (exclusively working day shifts, evening shifts or night shifts versus rotating shifts).

The second part addressed nurses’ opinion on barriers and facilitators to monitoring of NSOs. For this purpose, the statements from a previous study on quality indicators in Dutch ICUs were used (De Vos et al., 2010). These statements on barriers were based on the validated framework of Cabana et al. (1999) regarding behaviour change in health care, and included the following domains: (i) knowledge (awareness or familiarity); (ii) attitude (motivation); and (iii) behaviour (external factors, time and organizational issues). The facilitators were based on a literature review by Davies, Powell and Rushmer (2007) regarding health care professionals’ views on enablers for quality improvements. For the current study, an independent expert group (*n *= 3), consisting of a team leader with a background in ICU nursing, a person with a PhD with a background in ICU nursing, and a staff nurse with a scientific background, evaluated the face validity and content validity of these statements, as well as their relevance for nurses. Based on this expert feedback and on relevant literature (Cummings et al., 2010; McFadden, Stock & Gowen, 2015; Weston, 2010), the barrier statement “monitoring of quality indicators can be done without huge investments” was replaced with “nurse‐sensitive indicators offer opportunities to increase nursing autonomy” and the facilitator “pay‐for‐performance” was replaced with “support manager”, resulting in a questionnaire including 11 statements on barriers and 13 facilitators to the monitoring of NSOs. These items were scored on a 5 point Likert‐scale, ranging from “strongly disagree” (*1) ‐ “strongly agree” (*5). In addition, we added a self‐developed item to the questionnaire to assess which NSOs are considered by ICU nurses to be nurse‐sensitive. Results on the 4 point Likert‐scale, ranging from “strongly disagree” (*1) to “strongly agree” (*4) were used to extract proportions on the importance of the 18 predefined indicators. Various Dutch databases, including the dataset of the Dutch Health Care Inspectorate (IGZ), the Dutch National Society of Intensive Care Medicine (NVIC), and the Netherlands Centre of Excellence in Nursing (LEVV) were used to develop the list with NSOs.

In the third part of the questionnaire, the validated Dutch version of the questionnaire Essentials of Magnetism II (D‐EoM II) was used to explore nurses’ perception of their work environment. The internal consistency of the D‐EoM II showed an acceptable Cronbach's alpha of 0.92 for the entire scale, and 0.58 to 0.92 for the eight subscales. While one subscale showed a low Cronbach's alpha, the authors claimed that the correlations between the items of this subscale were high, and therefore they did not alter the subscale (De Brouwer et al., 2014). The D‐EoM II contains 58 statements and the EoM II was designed to assess the eight domains which are essential for a magnetic and healthy work environment: (i) working with clinically competent peers; (ii) support for education; (iii) collaborative nurse‐physician relationships; (iv) practice of clinical autonomy; (v) control of nursing practice; (vi) leadership and nurse manager support; (vii) patient‐centered cultural values; and (viii) adequacy of staffing (Kramer & Schmalenberg, 2004). These statements were scored on a 4 point Likert‐scale, ranging from “strongly disagree” (*1) ‐ “strongly agree” (*4).

### 2.4 Data analysis

First, descriptive statistics were used to characterize the study sample of responding ICU nurses. Second, nurses’ perception of barriers and facilitators were analysed using proportions on the 24 items. To calculate an overall mean score (MS) of the barrier domains of knowledge, attitude and behaviour, we used negative, neutral and positive formulated statements, including reverse‐order questions. A score less than 3 was considered as a negative overall result, indicating a need for improvement. Responses that were missing a value for one or more statements in a barrier domain resulted in the data for that domain being excluded from the data analysis. In addition, to explain differences in scores among subgroups, we used analysis of variance (ANOVA) with the overall mean scores on the domains as response variables and nurse characteristics as explanatory variables. Then, nurse characteristics were accounted for by involving all variables simultaneously in a multiple linear regression analysis. Dummy variables were created for the three units (Unit A, B and C). Multi‐collinearity was tested by means of the variance inflation factor (VIF) and tolerance value. Variables with a VIF >10 or a tolerance of <0.10 were suspected for multi‐collinearity and were excluded from further analysis (Stevens, 1992). Lastly, for each individual ICU the overall mean scores of the eight domains which considered as essential for a magnetic and healthy work environment were calculated, using negative and positive formulated statements. A score less than 2.5 indicated a negative result and a need for improvement. A *p*‐value of <0.05 was considered statistically significant. SPSS version 22 was used for quantitative analysis (IBM SPSS Statistics for Windows, Armonk, NY, USA: IBM Corp.).

### 2.5 Ethical consideration

Ethics approval for this study was granted by the hospitals’ Medical Ethical Review Commission (W13.030). The board of directors of each hospital involved in this study gave formal permission to conduct the study.

## 3 RESULTS

The overall response rate across the three ICUs was 45% (site range, 43%–46%), representing 126 ICU nurses. The majority of these respondents were female (78%), educated at least at the Bachelor's level (70%), working rotating shifts (87%) and working full‐time (62%). The median age was 41 years (IQR = 30–50), the median for nurses’ working experience was 20 years (IQR = 10–30), and for experience in the ICU the median was 11 years (IQR = 4–21) (Table 1).

**Table 1:** Baseline demographics of the study population

Nurse characteristics^a^	*N*	(%)
Responding nurses	126	(100)
Gender		
Male	28	(22.4)
Female	97	(77.6)
Education level		
Associate's degree	37	(29.6)
At least Bachelor's degree	88	(70.4)
Working shifts		
Regular shifts	16	(12.8)
Rotating shifts	109	(87.2)
Full‐time working		
Full‐time working	77	(61.6)
Part‐time working	48	(38.4)
Age, years	40.9 (±10.7)	
<40	58	(46.4)
40–49	36	(28.8)
≥50	31	(24.8)
Nursing experience, years	20.0 (±11.5)	
<10	30	(24.2)
10–19	31	(25.0)
≥20	63	(50.8)
ICU experience, years	13.0 (±10.1)	
5	37	(29.6)
5–14	37	(29.6)
≥15	51	(40.8)

a Missing values for gender, education level, working shifts, full‐time working, age, ICU experience (*N *=* *1) and nursing experience (*N *=* *2).

### 3.1 Barriers and facilitators to NSO monitoring

**Figure 1:**
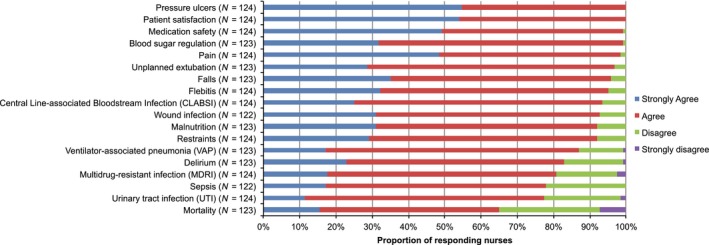
Nurse‐sensitivity of indicators, as perceived by ICU nurses

Figure 1 shows that the indicators pressure ulcers and patient satisfaction were fully perceived as nurse‐sensitive (100%), while mortality was not considered nurse‐sensitive by 35% (*n *=* *43) of respondents. Additionally, urinary tract infections (UTI), delirium, sepsis and multidrug‐resistant (MDR) infections were not perceived to be nurse‐sensitive by approximately 20% of respondents.

**Figure 2:**
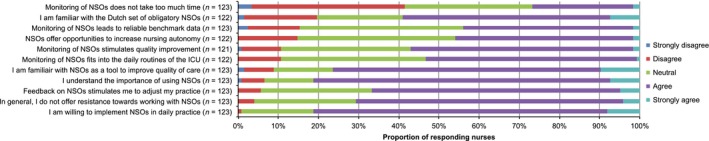
Barriers with regard to the monitoring of NSOs, as perceived by ICU nurses

As shown in Fig. 2, 42% (*n *=* *51) agreed that the monitoring of NSOs takes too much time (behaviour domain), nearly 20% (*n *=* *24) was not familiar with the mandatory set of NSOs as determined by the Dutch Health Care Inspectorate (knowledge domain), and 15% (*n *=* *19) did not agree that monitoring leads to reliable benchmark data (attitude domain).

**Figure 3:**
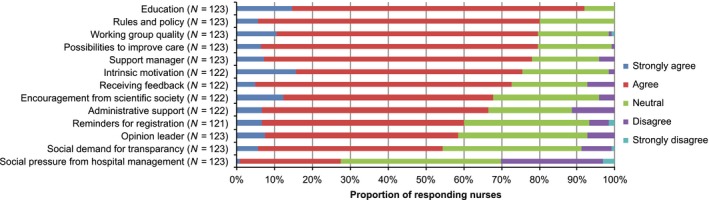
Facilitators with regard to the monitoring of NSOs, as perceived by ICU nurses

Figure 3 illustrates the perceived facilitators; nearly 92% (*n *=* *105) of nurses were in need of education about NSOs and 80% (*n *=* *98) agreed that clear rules and policies on NSOs in the unit are important facilitators. One‐third of the respondents mentioned that social pressure from the hospital management is ineffective as a facilitating factor.

### 3.2 Relationship with nurse characteristics and work environment

Collinearity statistics showed that age was interfering too much with other nurse characteristics (VIF = 13, tolerance = 0.08), and therefore age was excluded from further analyses. For the units combined, all domain scores regarding barriers were positive (MS ≥ 3); behavior (*M = *3.21, *SD *0.60), knowledge (*M *=* *3.60, *SD *0.78) and attitude (*M *=* *3.63, *SD *0.45). Subgroup analysis revealed that there were significant differences between units regarding the behaviour domain; one unit (unit B) had a negative and significantly lower behaviour related score (*M *=* *2.90, *SD *0.59; *p *<* *0.003), as compared to the other units (*M *=* *3.28, *SD *0.49; *M *=* *3.37, *SD *0.69). Further tests of differences in overall domain scores among subgroups showed a significantly higher score for the attitude domain in regular working nurses as compared to nurses working rotating shifts. Those working regular shifts scored 3.86 (*SD *0.40) versus those working rotating shifts who scored 3.60 (*SD *0.46). None of the other nurse characteristics were statistically significant related to the overall domain scores. The multiple linear regression analysis, as shown in Table 2, confirms that after adjusting for nurse characteristics, nurses in unit B gave a significant lower behaviour‐related score as compared to nurses in the other units (R^2^ = 0.15, F(8, 120) = 2.42, *p *=* *0.02).

**Table 2:** Multiple linear regression results for the barrier domains of knowledge, attitude and behaviour

	Knowledge		Attitude		Behaviour	
Nurse characteristics	Beta	*p*‐value	Beta	*p*‐value	Beta	*p*‐value
Unit A (versus unit B)	0.09	0.47	0.03	0.81	0.32	<0.01^a^
Unit C (versus unit B)	0.07	0.54	0.21	0.07	0.36	<0.01^a^
Female (versus male)	−0.17	0.10	0.04	0.68	0.03	0.74
Bachelor (versus Associate)	0.30	0.78	−0.04	0.69	0.12	0.23
Rotating (versus regular)	−0.05	0.67	−0.19	0.08	−0.20	0.05
Full‐time (versus part‐time)	0.02	0.83	0.04	0.73	−0.08	0.41
Nursing experience	−0.17	0.46	−0.01	0.96	−0.27	0.18
ICU experience	0.25	0.28	0.01	0.95	0.18	0.38

a Significant at *p *<* *0.05 level.

For the three units combined, the overall mean scores on the eight work environment domains were positive (MS ≥ 2.5). Nurses were most satisfied with adequacy of staffing (*M *=* *3.01, *SD *0.39) and least satisfied with control of practice (*M *=* *2.71, *SD *0.35). The only negative score related to work environment was unit B's ‘practice of clinical autonomy’; which was significantly lower (*M *=* *2.46, *SD *0.42; *p *<* *0.001) than the scores from other units for this same area (*M *=* *2.93, *SD * 0.22; *M *=* *2.93, *SD * 0.35).

## 4 DISCUSSION

This study aimed to investigate potential barriers and facilitators to monitoring of nurse‐sensitive outcomes (NSOs) from the perspective of nurses in Dutch intensive care units (ICUs), and to explore influential nurse characteristics and work environment factors. A major strength of this study is that we determined barriers and facilitators with regard to a wide range of NSOs, in contrast to previous studies focusing on one single NSO (El Hussein et al., 2014; Leistra et al., 2014; Strand & Lindgren, 2010; Wang & Tsai, 2010). As a result, we were able to draw more comprehensive conclusions about NSO monitoring by ICU nurses.

We found that all nurses agreed that pressure ulcers and patient satisfaction were clearly nurse‐sensitive indicators. Fewer nurses agreed regarding presumed NSOs, such as mortality, urinary tract infections, and sepsis. These findings contradicted those of Needleman et al. (2007) who referred to urinary tract infection and sepsis to be highly nurse‐sensitive. It is important to know how ICU nurses view NSOs, as those nurses who not perceive them as reliable and valid outcome measures of their work will be less likely to be motivated to adequately monitor these NSOs.

Another important finding was that lack of time was perceived as a major behaviour related issue in the monitoring of NSOs in ICUs. Besides the usual care practices, the administrative burden on nurses is increasingly present in the contemporary health care setting (De Vos et al., 2009). NSOs can be important indicators for the quality of care; however, in order to persuade nurses to behave accordingly, health care organizations need to place an emphasis on how monitoring NSOs relates to nurses’ regular duties and responsibilities, and that monitoring is not an unnecessary time‐consuming activity. One way in which this can be achieved is by determining the usefulness of NSOs in various types of units (Burston, Chaboyer & Gillespie, 2014). For example, specific NSOs, such as pressure ulcers and delirium frequently occur in patients admitted to critical care units, but are not as common in step‐down units involving patients with lower levels of complexity. As a result, nurses in critical care units should dedicate more time to monitoring these specific NSOs than non‐critical care units.

One reason for not screening NSOs is an ignorance on the part of nurses that screening for NSOs is part of their job requirement. For example, nearly 20% of nurses in the current sample were not familiar with the set of NSOs mandated by the Dutch Health Care Inspectorate. De Vos et al. (2010) reported that nurses in Dutch ICUs perceived higher levels of unfamiliarity with mandatory indicators than other health care professionals. Another study demonstrated that nurses in Magnet hospitals in the USA perceived lack of communication regarding mandatory NSOs as an important barrier to monitoring those NSOs as required (Beckel, Wolf, Wilson & Hoolahan, 2013). These knowledge related barriers are relatively easy to counter, and the most commonly described facilitators in this study, more education and clear policies, could stimulate NSO knowledge in ICUs and ideally improve the screening levels. The relevance of continuing education has been mentioned in previous studies investigating screening processes by health care professionals (Leistra et al., 2014).

In addition to barriers related to behaviour and knowledge, other factors identified as potentially contributing to suboptimal monitoring of NSOs were related to nurses’ attitudes. For example, 15% of nurses in our sample did not understand that NSO data could be utilized for benchmark purposes. This implies that simply informing nurses of the requirement to monitor NSOs may not be enough; in order to make a change, nurses need to understand how data related to NSOs is used by the local and national health care organizations. The abstract nature of attitude related barriers make them more difficult to overcome than knowledge related barriers, and changing a nurse's attitude often takes much longer than changing a nurse's level of education on NSOs. While attitude related barriers may prove more challenging than other barriers, they have a large impact on clinical outcomes, such as ventilation associated pneumonia, pressure ulcers and central line infections (Beeckman et al., 2007; Soh, Davidson, Leslie, DiGiacomo & Soh, 2013). In line with Baker et al.'s (2015) review of health professionals’ performance interventions, this study emphasizes that future interventions to improve nurses’ compliance with NSOs should be tailored to and focused on prospectively identified barriers; such as enhancing positive attitudes towards NSOs. This could be achieved by interactive learning and feedback, as previously reported by Pittet et al. (2000).

In line with previous NSO studies (De Vos et al., 2010; Wang & Tsai, 2010) various nurse characteristics, such as gender and educational level were included in the study analysis. Besides differences between regular versus rotating working nurses, we could not find any relevant associations with perceived barriers. Although the present study does not allow us to directly assess the specific contribution of work environment factors to nurses’ perception of barriers to monitoring NSOs, it did identify a potential link between nurses’ satisfaction with clinical autonomy and nurses’ perceived barriers. This is important, because satisfaction with work environments is relevant in relation to nursing processes. For example, studies on nursing care left undone showed that less favourable work environments are associated with higher levels of care left undone (Ausserhofer et al., 2014). Additionally, autonomy has been directly linked to both nurse outcomes (turnover, job satisfaction) as well as patient outcomes (patient safety, mortality) (Weston, 2010). Future studies should further investigate the role of work environment factors in a larger sample of ICUs, in order to test the study findings regarding potentially modifiable factors that may affect nursing processes and quality of care.

### 4.1 Limitations

Several study limitations occurred during the course of this study. These limitations concerned cross‐sectional data and as a result no causality could be demonstrated for the study findings. Another limitation is the generalizability of the results, since internationally a variety of NSOs are used to benchmark nursing care in hospitals. Delirium and malnutrition are mandatory NSOs in the Netherlands, whereas many other countries exclude these NSOs. Future empirical research should be performed consistently to determine the nurse‐sensitivity of indicators and their usefulness in different health care settings and countries. Although this study had an acceptable response rate of 45% (Baruch & Holtom, 2008), bias from non‐responders was another limitation in this study. This response rate is comparable to that of other survey studies focusing on critical care nurses (Cahill, Murch, Cook & Heyland, 2012) and the demographic characteristics of our sample resemble that of the full population of Dutch ICUs (Hansen, Van Velden & Hingstman, 2008).

## 5 CONCLUSIONS

NSOs are frequently used as indicators for the quality of nursing care in ICUs; however, various barriers exist to the appropriate monitoring of NSOs. This study contributes to the current literature by focusing on nurses, the health care professionals who have a key role in NSO utilization. Greater understanding of barriers and facilitators enables health care organizations to provide future tailored interventions aimed at optimally integrating NSOs into daily nursing practice. Enhancing nursing knowledge, behaviour and attitude towards the necessity of NSO monitoring is one way to increase nurses’ understanding of NSOs and NSO monitoring. Further research on work environment factors that potentially affect nursing processes in ICUs is needed in order to permanently improve and optimize nursing quality in these high‐intensity units.

## ACKNOWLEDGEMENT

We thank Anna van der Rhee and Renice Washington for checking the English language.

## AUTHORSHIP

All authors fulfil at least one of the criteria of authorship as specified in the “International Committee of Medical Journal Editors (ICMJE) Guidelines”, and approved the final version.

## AUTHOR CONTRIBUTIONS

DS contributed to the design and realization of the study, coordinated the data collection and analysis, and drafted and revised the manuscript. MV revised the manuscript critically for important intellectual content and analysis. DL, MK and MS contributed to the concept and design of the study, helped to draft the manuscript and revised the manuscript critically.

## CONFLICT OF INTEREST

No conflict of interest has been declared by the authors.

## PATIENT CONSENT

There was no need to seek patient consent, because no patients were involved in the study.
